# Cure for Drunkenness

**Published:** 1882-03

**Authors:** 


### CURE FOR DRUNKENNESS.

DR. JURIE, a prominent physician of Vienna, tells of two remarkable complete cures of dipsomania effected by him in an extraordinary manner. One of the cases was that of a habitual drunkard who was picked out of the gutter by the police, and was handed over to the doctor's treatment in the "Correction Hospital" for a period of fourteen days. The doctor at once ordered that every article of food or drink given him should receive a liberal addition of whisky of a not over-refined quality. Water, milk, soup, meat and vegetables were all treated in this way, and whisky was even infused into the air that he breathed, through saturation of the walls, floors and bedding. At first the man proclaimed himself highly satisfied with his treatment, and said he would always like to have such a sensible physician. The second day, however, he began to feel nausea, the third day he vomited immediately after eating, and thereafter not a meal was taken that was not followed by vomiting. From day to day he experienced increasing torment, and finally begged piteously for relief. The result was that at the end of two weeks, though much reduced in flesh, he was filled with such repugnance for strong drink that he was never able after to indulge in it again. The other case mentioned by Dr. Jurie was of a similar character, and was treated by him in the same way and with equal success.

